# Maintenance Risankizumab Sustains Induction Response in Patients with Crohn’s Disease in a Randomized Phase 3 Trial

**DOI:** 10.1093/ecco-jcc/jjad168

**Published:** 2023-10-05

**Authors:** Marc Ferrante, Peter M Irving, Maria T Abreu, Jeffrey Axler, Xiang Gao, Qian Cao, Toshimitsu Fujii, Astrid Rausch, Joana Torres, Ezequiel Neimark, Alexandra Song, Kori Wallace, Kristina Kligys, Sofie Berg, Xiaomei Liao, Qing Zhou, Jasmina Kalabic, Brian Feagan, Remo Panaccione

**Affiliations:** Department of Gastroenterology and Hepatology, University Hospitals Leuven, KU Leuven, Leuven, Belgium; IBD Centre, Guy’s and St Thomas’ NHS Foundation Trust, London, UK; University of Miami Miller School of Medicine, Miami, FL, USA; Toronto Digestive Disease Associates Inc., Vaughan, ON, Canada; Department of Gastroenterology, The Center for Inflammatory Bowel Disease, The Sixth Affiliated Hospital, Sun Yat-sen University, Guangzhou, Guangdong, China; Department of Gastroenterology, Sir Run Run Shaw Hospital, Zhejiang University School of Medicine, Hangzhou, Zhejiang, China; Department of Gastroenterology and Hepatology, Tokyo Medical and Dental University, Tokyo, Japan; Gastroenterology Department, British Hospital, Buenos Aires, Argentina; Division of Gastroenterology, Hospital Beatriz Ângelo, Loures, Portugal; Hospital da Luz, Lisbon, Portugal; Faculdade de Medicina, Universidade de Lisboa, Lisbon, Portugal; AbbVie Inc., North Chicago, IL, USA; AbbVie Inc., North Chicago, IL, USA; AbbVie Inc., North Chicago, IL, USA; AbbVie Inc., North Chicago, IL, USA; AbbVie Inc., North Chicago, IL, USA; AbbVie Inc., North Chicago, IL, USA; AbbVie Inc., North Chicago, IL, USA; AbbVie Deutschland GmbH & Co. KG, Ludwigshafen, Germany; University of Western Ontario, London, ON, Canada; Division of Gastroenterology and Hepatology, Cumming School of Medicine, University of Calgary, Calgary, AB, Canada

**Keywords:** Risankizumab, Crohn’s disease, durability

## Abstract

**Background and Aims:**

Durable clinical remission, endoscopic healing, and biomarker normalization are key treatment goals for Crohn’s disease. The selective anti-interleukin-23 p19 inhibitor risankizumab has demonstrated efficacy and safety in moderately to severely active Crohn’s disease. This post-hoc analysis of data from the pivotal risankizumab maintenance study assessed whether risankizumab maintenance therapy sustained the clinical and endoscopic outcomes achieved with risankizumab induction therapy.

**Methods:**

We evaluated 462 patients who achieved a clinical response to risankizumab intravenous induction treatment and were re-randomized to receive subcutaneous risankizumab 360 mg, subcutaneous risankizumab 180 mg, or placebo [withdrawal] every 8 weeks for 52 weeks in the randomized, controlled FORTIFY maintenance study. Maintenance of clinical, endoscopic, and biomarker endpoints at week 52 among patients who achieved these endpoints after 12 weeks of induction treatment was evaluated.

**Results:**

A significantly higher proportion of patients receiving maintenance treatment with risankizumab 360 or 180 mg compared with placebo [withdrawal] maintained Crohn’s Disease Activity Index remission [68.6%, 70.8%, vs 56.3%; *p* < 0.05], stool frequency/abdominal pain remission [69.2%, 64.1%, vs 50.5%; *p* < 0.01], endoscopic response [70.2%, 68.2%, vs 38.4%; *p* < 0.001], endoscopic remission [74.4%, 45.5%, vs 23.9%; *p* < 0.05], and Simple Endoscopic Score for Crohn’s Disease of 0–2 [65.5%, 36.7%, vs 21.9%]. Most patients [56.8–83.3%] who achieved normalized faecal calprotectin or C-reactive protein during induction sustained them with maintenance risankizumab.

**Conclusions:**

Subcutaneous risankizumab maintenance therapy results in durable improvement in clinical and endoscopic outcomes over 1 year in patients with moderately to severely active Crohn’s disease.

**Clinical trial registration number:**

NCT03105102.

## 1. Introduction

Crohn’s disease [CD] is a chronic, inflammatory disorder characterized by persistent or recurring inflammation, development of complications, and disability.^1^ While the introduction of anti-tumour necrosis factor therapies in clinical practice has brought major improvements in the management of CD, not all patients respond.^2^ Moreover, the use of these therapies is complicated by a high rate of immunogenicity development, leading to high rates of secondary loss of response, estimated to occur in 23–46% of patients over time.^2,3^ Patients who do not respond or lose response to biological therapies generally have diminished responses to subsequent lines of therapy.^4^ Often, loss of response leads to use of corticosteroid rescue therapy; prolonged use of corticosteroids is associated with increased morbidity and mortality.^5^ These challenges underline a need for new therapies with durable and sustained response.

It is undeniable that relieving symptoms of abdominal pain [AP] and increased stool frequency [SF] are critical therapeutic goals in CD. Indeed, improvement in health-related quality of life is highly correlated with decreased SF and AP.^6,7^ However, relief of symptoms is insufficient as a long-term treatment target. Absence of persistent endoscopic inflammation, measured by endoscopy, is associated with better long-term outcomes, including decreased rates of hospitalization, disease-related complications, and surgery.^8^ Accordingly, endoscopic healing measured by the Simple Endoscopic Score for Crohn’s Disease [SES-CD], restored health-related quality of life, and absence of disability are now considered to be the major long-term treatment goals for patients with CD according to the updated Selecting Therapeutic Targets in Inflammatory Bowel Disease recommendations.^8^

Risankizumab is a humanized immunoglobulin G1 monoclonal antibody that specifically inhibits interleukin-23 by binding to its p19 subunit.^9,10^ Phase 3 induction and maintenance trials conducted in patients with moderately to severely active CD have demonstrated that risankizumab provides symptom relief, improvement in endoscopic outcomes, and decreased inflammation relative to placebo, in addition to being well tolerated through 64 weeks.^11,12^ However, the durability of maintaining specific clinical and endoscopic outcomes achieved with 12-week induction therapy has not been well described in the literature. Herein, we report post-hoc analyses evaluating the effect of risankizumab maintenance therapy on the durability of clinical and endoscopic response and remission over a 1-year period.

## 2. Methods

### 2.1. Study design and treatment

ADVANCE [NCT03105128] and MOTIVATE [NCT03104413] were phase 3, randomized, double-blind, placebo-controlled studies in which patients with moderately to severely active CD received intravenous [IV] risankizumab [600 or 1200 mg] or placebo at baseline, week 4, and week 8 in a 12-week induction period. Clinical non-responders at week 12 were eligible to receive 12 weeks of risankizumab induction therapy during an extended 12-week treatment period. Patients achieving clinical response [defined as a ≥ 30% decrease in average daily SF and/or ≥ 30% decrease in average daily AP score, and both not worse than baseline of the induction study] in either the ADVANCE or MOTIVATE studies at week 12 or 24 were eligible to enrol in the FORTIFY study [NCT03105102]. FORTIFY was a phase 3, randomized, double-blind, placebo-controlled, 52-week maintenance withdrawal trial. Patients enrolled in the FORTIFY study were randomized 1:1:1 to receive subcutaneous [SC] risankizumab 180 mg, risankizumab 360 mg, or placebo [withdrawal from risankizumab] every 8 weeks for 52 weeks. Detailed descriptions of the ADVANCE, MOTIVATE, and FORTIFY study designs, patients, and procedures have been previously reported.^11,12^ Only methods relevant to this post-hoc analysis are described herein. All authors had access to the study data and reviewed and approved the final manuscript. This study was conducted in accordance with the International Conference on Harmonization guidelines and the Declaration of Helsinki.

### 2.2 Patients

Eligible patients were aged 16‒80 years and had moderately to severely active CD. Patients were classified as either a biologic inadequate responder (Bio-IR [i.e. had intolerance and/or inadequate response to one or more biologic therapies]) or a non-biologic inadequate responder (non-Bio-IR [i.e. had previous intolerance and/or inadequate response to conventional therapies, such as immunosuppressants or corticosteroids]). Patients who had previously received biologic therapy but stopped for reasons other than inadequate response and/or intolerance [e.g. change in insurance coverage] were considered non-Bio-IR.

### 2.3 Assessments

The effect of risankizumab on durability of response was evaluated based on maintenance of clinical, endoscopic, and/or biomarker outcomes at week 52 among patients who achieved the same outcome after 12 weeks of risankizumab induction therapy [week 0 of maintenance], regardless of induction dose. In addition, subgroup analyses were performed by induction baseline Bio-IR status.

#### 2.3.1. Clinical assessments

Symptom improvement durability was assessed using the proportion of patients achieving clinical remission at week 52, either defined by Crohn’s Disease Activity Index [CDAI < 150] or SF/AP [average daily SF ≤ 2.8 and average daily AP score ≤ 1 and both not worse than baseline] among patients who achieved the same outcome at the end of risankizumab induction therapy, regardless of induction dose.

#### 2.3.2. Endoscopic assessments

The durability of endoscopic outcomes at week 52 [endoscopic response, endoscopic remission, and SES-CD of 0–2] was assessed among patients who achieved the same outcome at the end of 12-week risankizumab induction therapy [week 0 of maintenance]. A group of blinded central readers assessed SES-CD for all efficacy analyses; total SES-CD was scored by a single blinded central reader for each patient, and, where possible, the same central reader who assessed the screening endoscopy video for a patient was assigned to read all subsequent videos for that patient. Endoscopic response was defined as > 50% reduction in SES-CD from baseline or, for patients with isolated ileal disease and an SES-CD of 4 at baseline, ≥ 2-point reduction from baseline. Endoscopic remission was defined as SES-CD ≤ 4 and ≥ 2-point reduction from baseline with no subscore > 1 in any individual variable.

#### 2.3.3. Biomarker assessments

The durability of biomarker normalization was assessed based on the proportion of patients maintaining a faecal calprotectin [FCP] concentration of ≤ 250 mg/kg or a high-sensitivity C-reactive protein [hs-CRP] concentration of ≤ 5 mg/L at week 52 among those who had elevated biomarker levels at the baseline of induction and normalized biomarker levels at the end of risankizumab induction therapy [week 0 of maintenance].

#### 2.3.4. Composite assessments

The durability of composite endpoints incorporating both clinical and either endoscopic or biomarker endpoints was evaluated at week 52 among patients who achieved the same outcomes at the end of risankizumab induction therapy. Composite endpoints included [1] CDAI clinical remission plus endoscopic remission [deep remission], [2] SF/AP clinical remission plus endoscopic remission, [3] maintenance of an FCP concentration of ≤ 250 mg/kg plus CDAI clinical remission, [4] maintenance of an FCP concentration of ≤ 250 mg/kg plus SF/AP remission, [5] maintenance of an hs-CRP concentration of ≤ 5 mg/L plus CDAI clinical remission, and [6] maintenance of an hs-CRP concentration of ≤ 5 mg/L plus SF/AP clinical remission.

#### 2.3.5. Continuous assessments

In addition to the categorical endpoints listed above, the continuous endpoints of CDAI, SF, AP, and SES-CD were assessed from induction baseline to week 52 of maintenance therapy as supportive analyses.

### 2.4. Statistical analysis

Patients included in the analyses had an eligible SES-CD of ≥ 6 [≥ 4 for isolated ileal disease] at baseline of the induction study, received IV risankizumab for only one period of 12 weeks in the induction study, and received at least one dose of study drug in the FORTIFY study. Categorical endpoints were analysed using non-responder imputation incorporating multiple imputation for missing values due to COVID-19 [NRI-C]; continuous endpoints were analysed using a mixed-effects model with repeated measures.

## 3. Results

### 3.1. Patients

A total of 462 patients responding to IV risankizumab were re-randomized to receive SC risankizumab 360 mg [*n* = 141], risankizumab 180 mg [*n* = 157], or placebo [withdrawal; *n *= 164] and were included in this analysis. Patient characteristics at baseline and week 0 of maintenance have been previously described.^12^ The study was completed by 412 [89.2%] patients, with the most frequent primary reason for study discontinuation being lack of efficacy (360 mg, 4.3%; 180 mg, 3.2%; placebo [withdrawal], 5.5%).

### 3.2. Efficacy assessments at week 52

**Figure 1.**
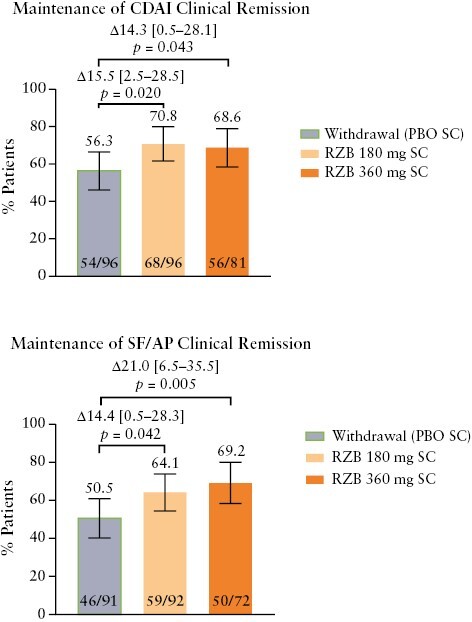
Maintenance of CDAI clinical remission and SF/AP clinical remission at week 52 among patients with the same outcome at the end of the induction period. Error bars represent the lower and upper bounds of the 95% confidence interval [95% CI]. Values above brackets are the adjusted treatment difference [95% CI]. AP, abdominal pain; CDAI, Crohn’s Disease Activity Index; PBO, placebo; RZB, risankizumab, SC, subcutaneous; SF, stool frequency.

Patients who achieved CDAI clinical remission at the end of induction were more likely to achieve clinical remission at week 52 than patients who did not achieve CDAI clinical remission at the end of induction [odds ratios: 4.42, risankizumab 360 mg; 5.37, risankizumab 180 mg; Supplementary Table 1]. Among 273 patients with CDAI clinical remission at week 0 of the maintenance study, 56 of 81 [68.6%] patients in the risankizumab 360 mg group and 68 of 96 [70.8%] patients in the risankizumab 180 mg group vs 54 of 96 [56.3%] patients maintained remission at week 52 in the placebo [withdrawal] group [360 mg, *p *= 0.043; 180 mg, *p *= 0.020; Figure 1). Similarly, patients who achieved SF/AP clinical remission at the end of induction were more likely to achieve clinical remission at week 52 than those who did not achieve SF/AP clinical remission at the end of induction [odds ratios: 4.26, risankizumab 360 mg; 6.51, risankizumab 180 mg; Supplementary Table 1]. Among 255 patients with SF/AP clinical remission at week 0 of the maintenance study, 50 of 72 [69.2%] patients and 59 of 92 [64.1%] patients vs 46 of 91 [50.5%] patients maintained remission at week 52 in the risankizumab 360 mg and 180 mg vs placebo [withdrawal] groups, respectively [360 mg, *p* = 0.005; 180 mg, *p* = 0.042; Figure 1].

**Figure 2.**
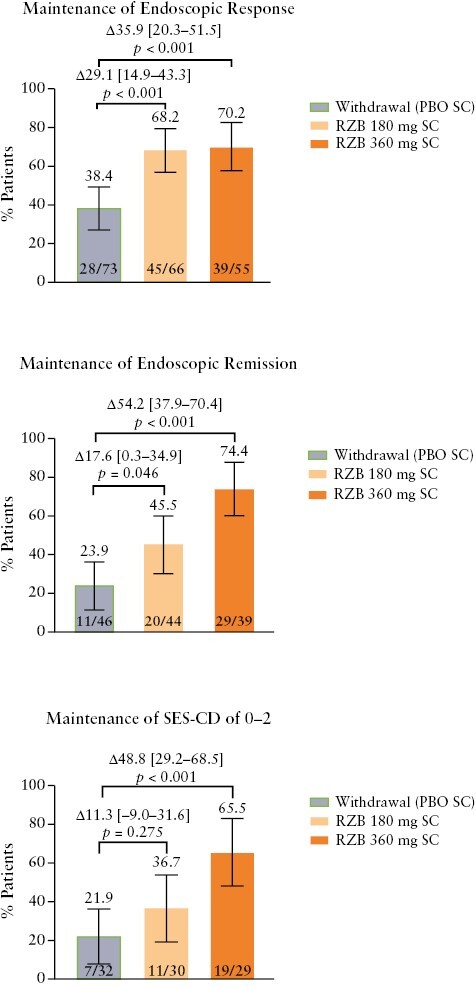
Maintenance of endoscopic response, endoscopic remission, and SES-CD of 0–2 at week 52 among patients with the same outcome at the end of the induction period. Error bars represent the lower and upper bounds of the 95% confidence interval [95% CI]. Values above brackets are the adjusted treatment difference [95% CI]. PBO, placebo; RZB, risankizumab; SC, subcutaneous; SES-CD, Simple Endoscopic Score for Crohn’s Disease.

Endoscopic outcomes were also sustained in patients receiving maintenance risankizumab therapy. Patients who achieved endoscopic response at the end of induction were more likely to achieve endoscopic response at week 52 than patients who did not achieve endoscopic response at the end of induction [odds ratios: 4.89, risankizumab 360 mg; 4.58, risankizumab 180 mg; Supplementary Table 1]. Among 194 patients with endoscopic response at week 0 of the maintenance study, 39 of 55 [70.2%] patients and 45 of 66 [68.2%] patients vs 28 of 73 [38.4%] patients maintained endoscopic response at week 52 in the risankizumab 360 mg and 180 mg vs placebo [withdrawal] groups, respectively [*p* < 0.001 for both; Figure 2]. Among patients who achieved endoscopic response at week 0 of maintenance, 35 of 55 [63.8%] patients and 32 of 66 [48.5%] patients vs 17 of 73 [23.3%] patients achieved endoscopic remission at week 52 in the risankizumab 360 mg and 180 mg vs placebo [withdrawal] groups, respectively [360 mg, *p < *0.001; 180 mg, *p *= 0.001). Both doses of risankizumab were more effective than placebo [withdrawal] for sustaining achievement of the more stringent endpoints of endoscopic remission and SES-CD of 0–2, with numerically higher outcomes for patients receiving risankizumab 360 mg relative to 180 mg. Among 129 patients who achieved endoscopic remission at week 0 of the maintenance study, this outcome was maintained at week 52 by 29 of 39 [74.4%] patients and 20 of 44 [45.5%] patients vs 11 of 46 [23.9%] patients in the risankizumab groups vs placebo [withdrawal] [360 mg, *p < *0.001; 180 mg, *p *= 0.046]. In the 91 patients with an SES-CD of 0–2 at week 0 of the maintenance study, an SES-CD of 0–2 was maintained by 19 of 29 [65.5%] patients [risankizumab 360 mg] and 11 of 30 [36.7%] patients [risankizumab 180 mg] vs 7 of 32 [21.9%] patients (placebo [withdrawal]; 360 mg, *p* < 0.001; 180 mg, *p* = 0.275; Figure 2). Endoscopic images from four patients exemplify endoscopic improvements over time in patients who responded to 12-week IV risankizumab induction and received either risankizumab 180 or 360 mg SC maintenance therapy or placebo [withdrawal of risankizumab; Supplementary Figure 1].

**Figure 3.**
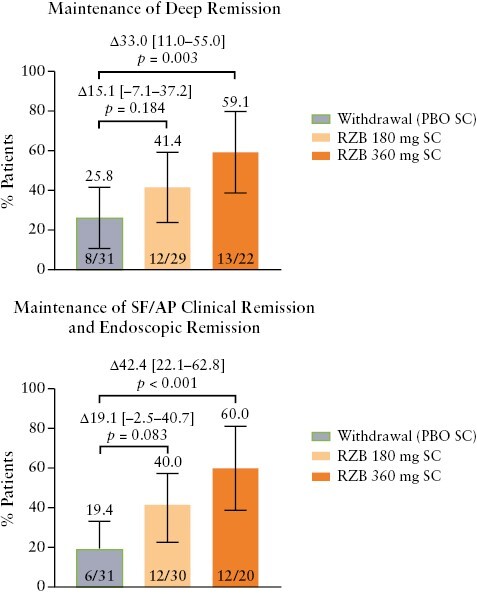
Maintenance of deep remission [CDAI clinical remission plus endoscopic remission] and SF/AP clinical remission plus endoscopic remission at week 52 among patients with the same outcome at the end of the induction period. Error bars represent the lower and upper bounds of the 95% confidence interval [95% CI]. Values above brackets are the adjusted treatment difference [95% CI]. AP, abdominal pain; CDAI, Crohn’s Disease Activity Index; PBO, placebo; RZB, risankizumab, SC, subcutaneous; SF, stool frequency.

Among patients who achieved composite outcomes at the end of induction therapy, these outcomes were maintained by higher proportions of patients receiving risankizumab maintenance therapy vs placebo [withdrawal]; outcomes were significantly different between the risankizumab 360 mg and placebo arms [Figure 3]. Risankizumab maintenance therapy led to durable improvement of clinical and endoscopic outcomes among patients who achieved these endpoints at the end of induction, regardless of Bio-IR status, though response rates for clinical outcomes were numerically higher in the non-Bio-IR subgroup. At week 52, CDAI clinical remission and SF/AP clinical remission, individually, were maintained by most patients receiving risankizumab 360 and 180 mg, regardless of Bio-IR status [Supplementary Figure 2]. Endoscopic response was maintained by the majority of patients receiving either dose of risankizumab, regardless of Bio-IR status [Supplementary Figure 3]. For the more stringent endpoints of endoscopic remission and SES-CD of 0–2, a dose–response relationship was observed, with the highest durable response rates observed in the risankizumab 360 mg group, regardless of Bio-IR status [Supplementary Figure 3]. A similar dose–response pattern was observed for the composite endpoints of deep remission and SF/AP clinical remission plus endoscopic remission, regardless of Bio-IR status [Supplementary Figure 4].

**Figure 4.**
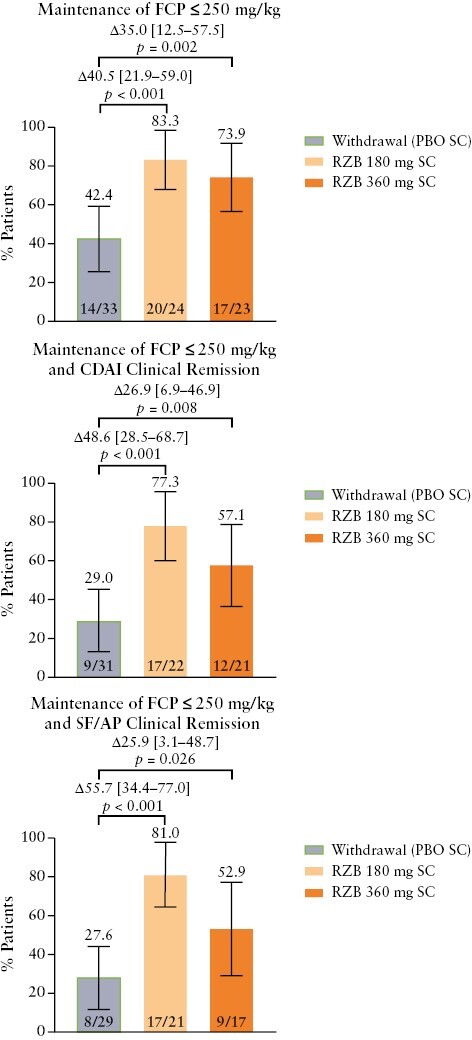
Maintenance of FCP ≤ 250 mg/kg, maintenance of FCP ≤ 250 mg/kg and CDAI clinical remission, and maintenance of FCP ≤ 250 mg/kg and SF/AP clinical remission at week 52 among patients who had elevated FCP at baseline and achieved the same outcome at the end of the induction period. Error bars represent the lower and upper bounds of the 95% confidence interval [95% CI]. Values above brackets are the adjusted treatment difference [95% CI]. AP, abdominal pain; CDAI, Crohn’s Disease Activity Index; FCP, faecal calprotectin; PBO, placebo; RZB, risankizumab, SC, subcutaneous; SF, stool frequency.

Among patients with an elevated FCP concentration at induction baseline who achieved FCP ≤ 250 mg/kg at the end of induction [*n* = 80], most patients receiving either dose of risankizumab sustained achievement of FCP ≤ 250 mg/kg at week 52, regardless of dose [360 mg, 73.9%, *p *= 0.002; 180 mg, 83.3%, *p < *0.001], compared with the placebo [withdrawal] group [42.4%; Figure 4]. Similar patterns were observed for the composite endpoints of maintenance of FCP ≤ 250 mg/kg plus CDAI clinical remission and maintenance of FCP ≤ 250 mg/kg plus SF/AP clinical remission [Figure 4].

**Figure 5.**
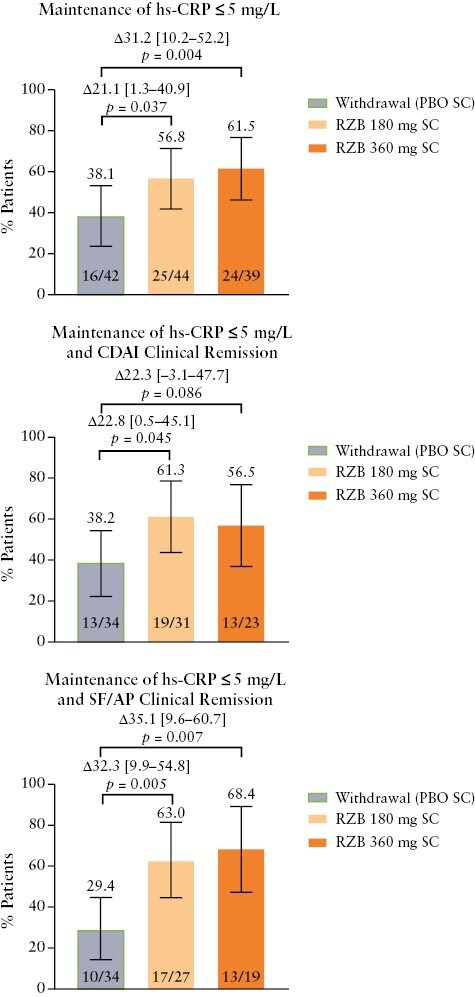
Maintenance of hs-CRP concentration of ≤ 5 mg/L, maintenance of hs-CRP concentration of ≤ 5 mg/L and CDAI clinical remission, and maintenance of hs-CRP concentration of ≤ 5 mg/L and SF/AP clinical remission at week 52 among patients who had elevated hs-CRP concentrations at baseline and achieved the same outcome at the end of the induction period. Error bars represent the lower and upper bounds of the 95% confidence interval [95% CI]. Values above brackets are the adjusted treatment difference [95% CI]. AP, abdominal pain; CDAI, Crohn’s Disease Activity Index; hs-CRP, high-sensitivity C-reactive protein; PBO, placebo; RZB, risankizumab, SC, subcutaneous; SF, stool frequency.

Among patients with elevated hs-CRP concentrations at induction baseline who achieved hs-CRP concentration of ≤ 5 mg/L at the end of induction [*n* = 125], most patients receiving either dose of risankizumab sustained achievement of hs-CRP concentration of ≤ 5 mg/L at week 52 [360 mg, 61.5%, *p* = 0.004; 180 mg, 56.8%, *p* = 0.037) vs patients in the placebo [withdrawal] group [38.1%; Figure 5]. Similar patterns were observed for the composite endpoints of maintenance of hs-CRP concentration of ≤ 5 mg/L plus CDAI clinical remission and maintenance of hs-CRP concentration of ≤ 5 mg/L plus SF/AP clinical remission [Figure 5].

**Figure 6.**
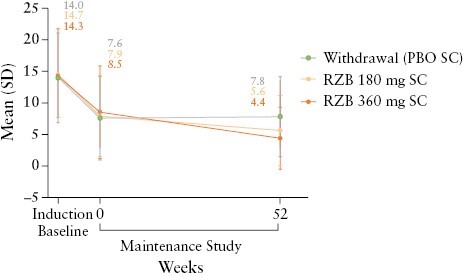
Mean SES-CD over time. Error bars represent the lower and upper bounds of the 95% confidence interval. PBO, placebo; RZB, risankizumab, SC, subcutaneous; SES-CD, Simple Endoscopic Score for Crohn’s Disease.

Starting from induction baseline, the results over time for the FORTIFY study population were generally consistent with and supportive of patterns observed for maintenance of induction study responses. While mean CDAI generally decreased over time during the maintenance study, a smaller reduction was observed for patients in the placebo [withdrawal] group compared with the risankizumab groups at week 52 [Supplementary Figure 5]. The SF and AP scores were also generally sustained near post-induction therapy levels through week 52 across groups [Supplementary Figure 5]. Correspondingly, clinical remission rates were generally maintained through week 24 among the overall FORTIFY study population [Supplementary Figure 6] but tended to decrease after week 24 in the placebo [withdrawal] group. Additionally, mean SES-CD decreased in the FORTIFY study population following risankizumab induction therapy; however, further decreases were noted over time for those patients receiving risankizumab maintenance therapy, while increases in mean SES-CD occurred in the placebo [withdrawal] group from the end of induction to week 52 [Figure 6].

### 3.3. Safety

Risankizumab was generally well tolerated during the induction and maintenance periods of the study, and no new safety signals were identified. Detailed safety information for both study periods was previously reported.^11,12^

## 4. Discussion

Symptomatic control in patients with CD is key in restoring quality of life; reducing SF and AP are important short-term treatment goals. However, endoscopic inflammation is implicated in worse outcomes, even among patients who have achieved clinical remission.^8^ The updated Selecting Therapeutic Targets in Inflammatory Bowel Disease recommendations identified endoscopic healing, improved quality of life, and absence of disability as suitable long-term treatment targets.^8^

Secondary loss of response also remains a concern for long-term treatment of CD, as patients with inadequate response to a biologic therapy generally have worse outcomes compared with those for whom a biologic therapy has not failed. While there are several biologic therapies being investigated as treatments for moderately to severely active CD, there remains a need for therapies with durable response, and relatively few reports of maintained symptomatic control and endoscopic healing exist in the literature.^13^

Intravenous risankizumab provides early disease control in patients with moderately to severely active CD as induction therapy.^11^ As maintenance therapy, SC risankizumab is effective at symptom control, improving endoscopic outcomes, and normalizing inflammatory biomarkers through week 52.^12^ Results of this post-hoc analysis show that most patients who achieve clinical and endoscopic outcomes after 12 weeks of risankizumab IV induction sustain those same outcomes with risankizumab maintenance therapy for up to an additional 52 weeks. For many patients with endoscopic response at maintenance baseline who received continuous risankizumab, endoscopic remission was achieved by week 52, with a numerically higher rate for patients receiving risankizumab 360 mg compared with patients receiving risankizumab 180 mg. In contrast, most patients in the withdrawal group [i.e. patients who responded to risankizumab induction therapy and had risankizumab withdrawn] lost endoscopic response by week 52; the loss of endoscopic response often precedes loss of symptomatic control.^14^ Furthermore, a higher proportion of patients receiving either risankizumab dose maintained normalization of biomarkers vs those who received placebo [withdrawal]. Though the durability of clinical response is relatively high, even in the withdrawal group, these results, particularly those for the loss of endoscopic and biomarker outcomes, highlight the importance of continuing risankizumab therapy to maintain disease control.

In this analysis, while symptomatic control responses were similar, regardless of risankizumab dose, for the more stringent endoscopic and composite endpoints, a higher proportion of patients receiving risankizumab 360 mg SC sustained response compared with patients receiving risankizumab 180 mg SC. This may suggest that the higher maintenance dose is more suitable for long-term treatment of CD.

This post-hoc analysis is the first to specifically evaluate the durability of clinical and endoscopic responses after they were achieved with risankizumab induction therapy. To our knowledge, this study also reports the first data on long-term maintenance of endoscopic response among non-Bio-IR patients. The interpretations of these results are limited by the post-hoc nature of the analysis [which precluded the calculation of sample sizes for statistical significance], the small patient populations [particularly in the biomarker analyses], and the long-lasting effects of risankizumab, which may have improved outcomes in the re-randomized induction responder placebo [withdrawal] group compared with a true placebo.

In conclusion, this analysis demonstrates the durable clinical and endoscopic benefits of risankizumab maintenance therapy for patients who responded to 12 weeks of induction therapy. These results also highlight the importance of continuing maintenance treatment to sustain the benefits of risankizumab treatment for signs and symptoms of moderately to severely active CD.

## Supplementary Material

jjad168_suppl_Supplementary_Material

## Data Availability

AbbVie is committed to responsible data sharing regarding the clinical trials it sponsors. This includes access to anonymized individual and trial-level data [analysis data sets], as well as other information [e.g. protocols, clinical study reports, or analysis plans], as long as the trials are not part of an ongoing or planned regulatory submission. This includes requests for clinical trial data for unlicensed products and indications. These clinical trial data can be requested by any qualified researchers who engage in rigorous, independent scientific research, and will be provided following review and approval of a research proposal and statistical analysis plan and execution of a data sharing agreement. Data requests can be submitted at any time after approval in the United States and Europe and after acceptance of this manuscript for publication. The data will be accessible for 12 months, with possible extensions considered. For more information on the process or to submit a request, visit the following link: https://vivli.org/ourmember/abbvie/ then select ‘Home’.

## Notes

**Conference Presentation**

The results reported in this publication were presented at the 17th Congress of European Crohn’s and Colitis Organization [ECCO], February 16–19, 2022, Virtual. The following abstracts are published in a supplementary issue of *J Crohns Colitis:*

Ferrante M, Cao Q, Fujii T, et al. Patients with moderate to severe Crohn’s disease with and without prior biologic failure demonstrate improved endoscopic outcomes with risankizumab: results from phase 3 induction and maintenance trials. *J Crohns Colitis* 2022;**16**[Suppl 1]:i027–i028. OP25.

Irving PM, Abreu M, Axler J, et al. Risankizumab maintenance therapy results in sustained improvements in endoscopic outcomes in patients with moderate to severe Crohn’s disease: post hoc analysis from the phase 3 study FORTIFY. *J Crohns Colitis* 2022;**16**[Suppl 1]:i127–i128. DOP84.

D’Haens G, Beaton M, Bossuyt P, et al. Patients with moderate to severe Crohn’s disease with and without prior biologic failure demonstrated improved clinical outcomes with risankizumab: results from phase 3 induction and maintenance trials. *J Crohns Colitis* 2022;**16**[Suppl 1]:i491. P544.
