# Supplementary material for: Maintenance Risankizumab Sustains Induction Response in Patients with Crohn’s Disease in a Randomized Phase 3 Trial
Source: J Crohns Colitis. 2023 Oct 5;18(3):416–23. doi: 10.1093/ecco-jcc/jjad168 (PMC10906949; doi:10.1093/ecco-jcc/jjad168)
Supplement: jjad168_suppl_Supplementary_Material [file jjad168_suppl_supplementary_material.pdf]

Supplemental Online Content

Ferrante M, Irving PM, Abreu MT, et al. Maintenance Risankizumab Sustains Induction Response in Patients with Crohn's Disease in a Randomized Phase 3 Trial

Supplementary Table 1. Odds ratios for achievement of key outcomes at week 52 of risankizumab maintenance treatment based on achievement of those outcomes at the end of induction.

Supplementary Figure 1. Endoscopic images over time.

Supplementary Figure 2. Maintenance of CDAI clinical remission and SF/AP clinical remission at week 52 by Bio-IR status.

Supplementary Figure 3. Maintenance of endoscopic response, endoscopic remission, and SES-CD 0–2 remission at week 52 by Bio-IR status.

Supplementary Figure 4. Maintenance of deep remission and SF/AP clinical remission plus endoscopic remission at week 52 by Bio-IR status.

Supplementary Figure 5. Mean CDAI, SF, and AP over time.

Supplementary Figure 6. Clinical remission over time in the maintenance study.

Supplementary Table 1. Odds ratios for achievement of key outcomes at week 52 of risankizumab maintenance treatment based on achievement of those outcomes at the end of induction.

	RZB 180 mg		RZB 360 mg	
**Outcome**	**Odds Ratio (95% CI)**	***p*-value**	**Odds Ratio (95% CI)**	***p*-value**
CDAI clinical remission	5.37 (2.67–10.79)	<0.001	4.42 (2.16–9.05)	<0.001
SF/APS clinical remission	6.51 (3.14–13.50)	<0.001	4.26 (2.11–8.61)	<0.001
Endoscopic response	4.58 (2.32–9.05)	<0.001	4.89 (2.35–10.15)	<0.001

From logistical regression model with week 0 outcome status as a fixed factor in the model. Status of “no” used as the reference group. APS, abdominal pain score; CDAI, Crohn’s Disease Activity Index; RZB, risankizumab; SF, stool frequency.

Supplementary Figure 1. Endoscopic images over time.

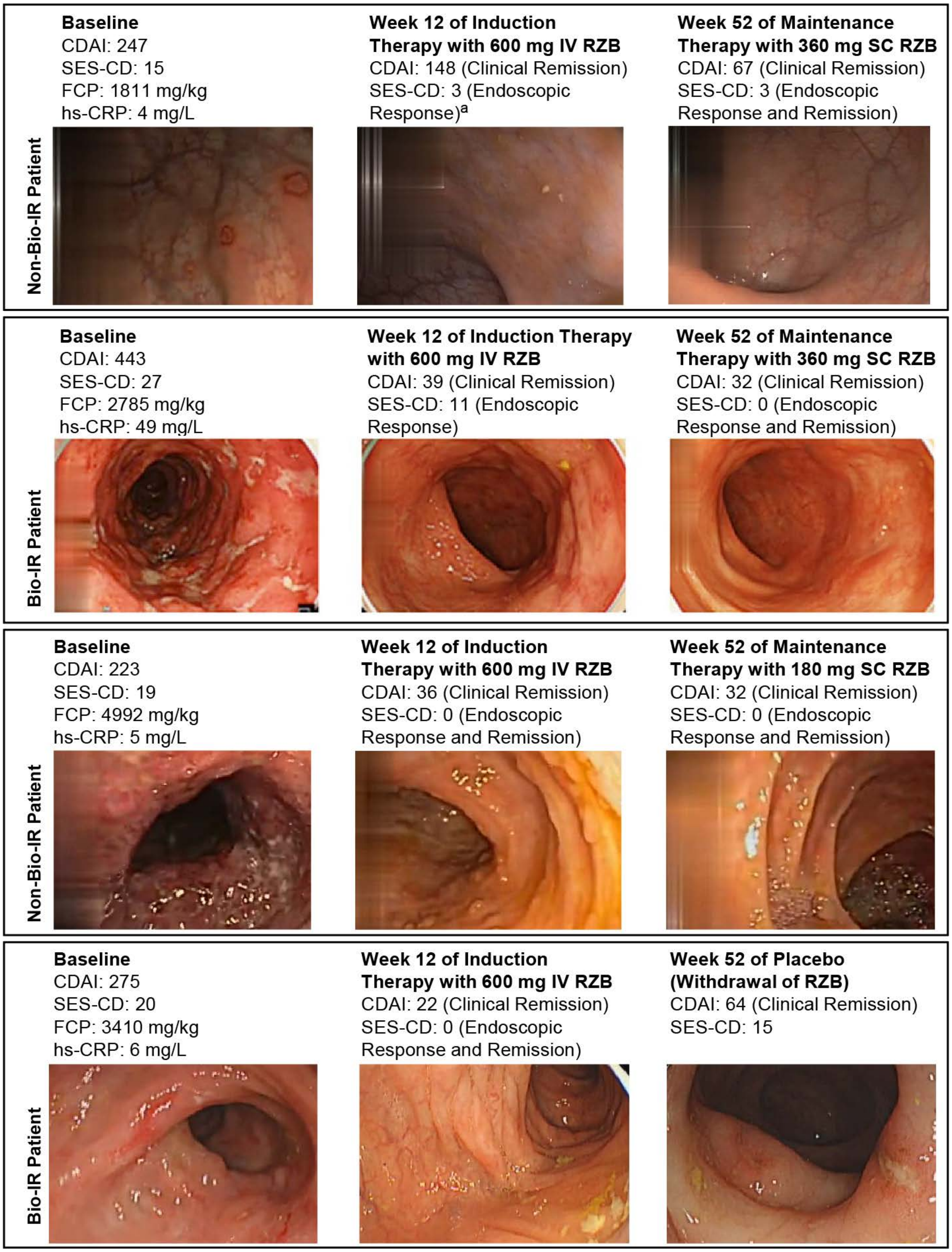

[a]While the patient achieved an SES-CD of 3 at week 12 of induction, they did not achieve endoscopic remission due to having a subscore >1 in a segment. At week 52 of maintenance, the patient achieved an SES-CD of 3 and endoscopic remission, due to having no subscore >1 in any segment.

Row 1: A patient without prior biologic failure who responded to 12 weeks of risankizumab 600 mg IV induction therapy and received 52 weeks of risankizumab 360 mg SC maintenance therapy; the patient maintained clinical remission and endoscopic response and gained endoscopic remission at week 52.

Row 2: A patient with prior biologic failure who responded to 12 weeks of risankizumab 600 mg IV induction therapy and received 52 weeks of risankizumab 360 mg SC maintenance therapy; the patient maintained clinical remission and endoscopic response and gained endoscopic remission at week 52.

Row 3: A patient without prior biologic failure who responded to 12 weeks of risankizumab 600 mg IV induction therapy and received 52 weeks of risankizumab 180 mg SC maintenance therapy; the patient maintained clinical remission, endoscopic response, and endoscopic remission at week 52.

Row 4: A patient with prior biologic failure who responded to 12 weeks of risankizumab 600 mg IV induction therapy and received 52 weeks of placebo [withdrawn from risankizumab]; the patient maintained clinical remission at week 52, but bowel inflammation returned after withdrawal of risankizumab and the patient lost endoscopic response and endoscopic remission at week 52.

AP, abdominal pain; CDAI, Crohn’s Disease Activity Index; IV, intravenous; SES-CD, Simple Endoscopic Score for Crohn’s Disease; SF, stool frequency; SC, subcutaneous.

Supplementary Figure 2. Maintenance of CDAI clinical remission and SF/AP clinical remission at week 52[a] by Bio-IR status.

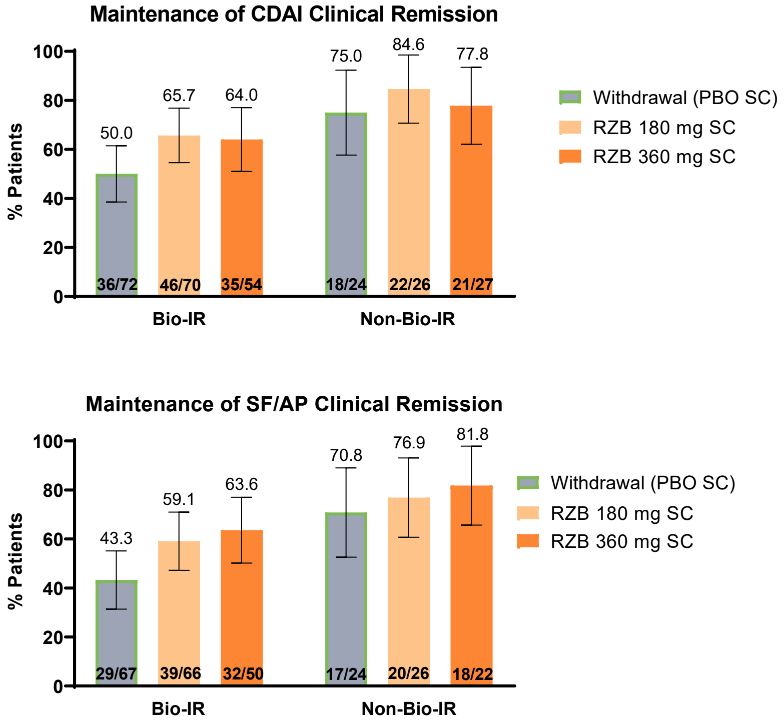

[a]Among patients with the same outcome at the end of the induction period. Error bars represent the lower and upper bounds of the 95% confidence interval. AP, abdominal pain; Bio-IR, intolerance and/or inadequate response to biologic therapies; CDAI, Crohn's Disease Activity Index; Non-Bio-IR, previous intolerance and/or inadequate response to conventional therapies; PBO, placebo; RZB, risankizumab, SC, subcutaneous; SF, stool frequency.

Supplementary Figure 3. Maintenance of endoscopic response, endoscopic remission, and SES-CD 0–2 remission at week 52[a] by Bio-IR status.

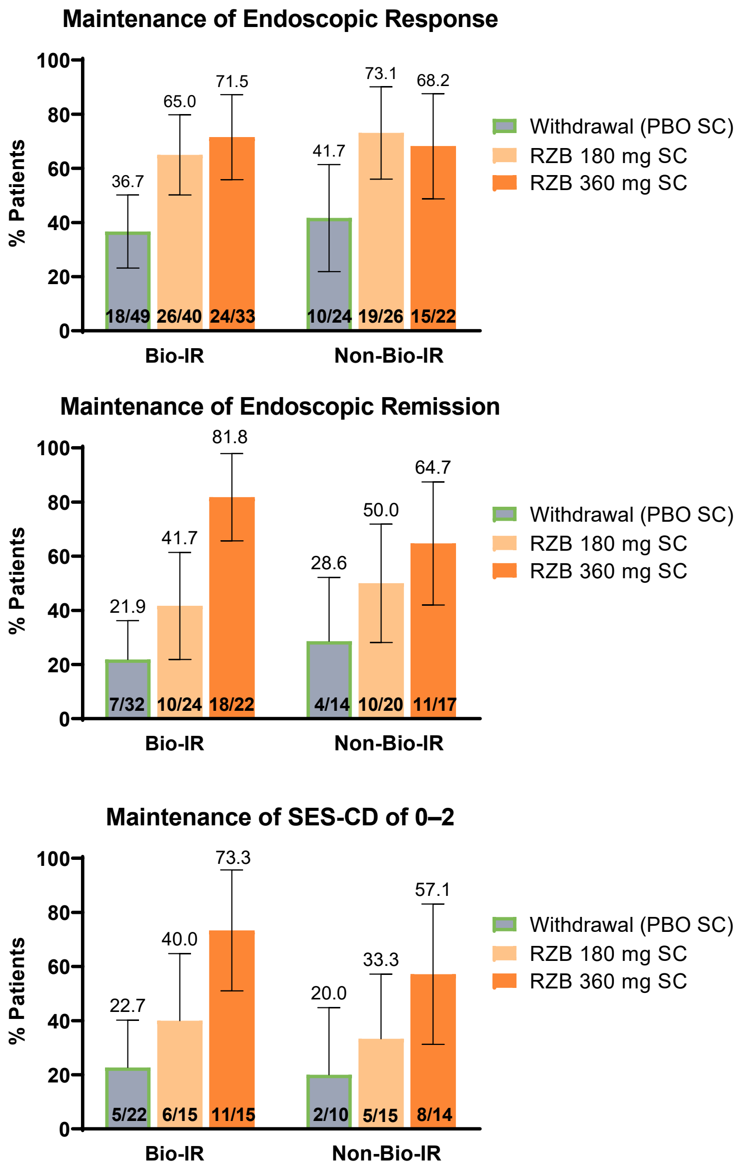

[a]Among patients with the same outcome at the end of the induction period. Error bars represent the lower and upper bounds of the 95% confidence interval.
Bio-IR, intolerance and/or inadequate response to biologic therapies;
Non-Bio-IR, previous intolerance and/or inadequate response to conventional therapies;
PBO, placebo; RZB, risankizumab, SC, subcutaneous; SES-CD, Simple Endoscopic Score for Crohn's Disease.

Supplementary Figure 4. Maintenance of deep remission and SF/AP clinical remission plus endoscopic remission at week 52[a] by Bio-IR status.

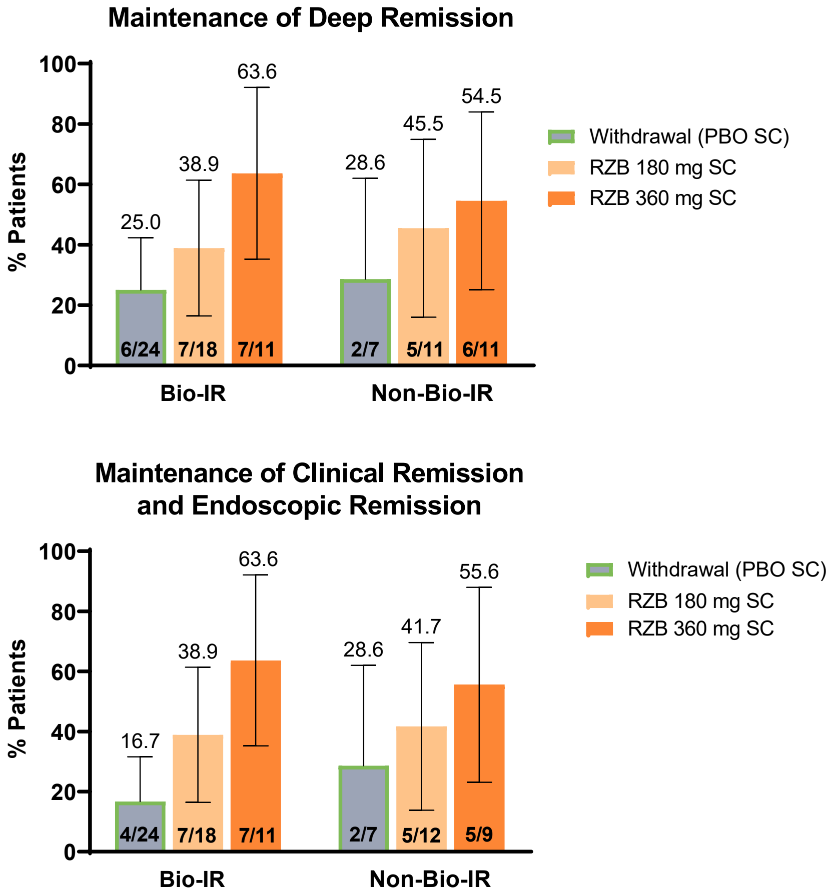

[a]Among patients with the same outcome at the end of the induction period. Error bars represent the lower and upper bounds of the 95% confidence interval. AP, abdominal pain; Bio-IR, intolerance and/or inadequate response to biologic therapies; Non-Bio-IR, previous intolerance and/or inadequate response to conventional therapies; PBO, placebo; RZB, risankizumab, SC, subcutaneous; SF, stool frequency.

Supplementary Figure 5. Mean CDAI, SF, and AP over time.

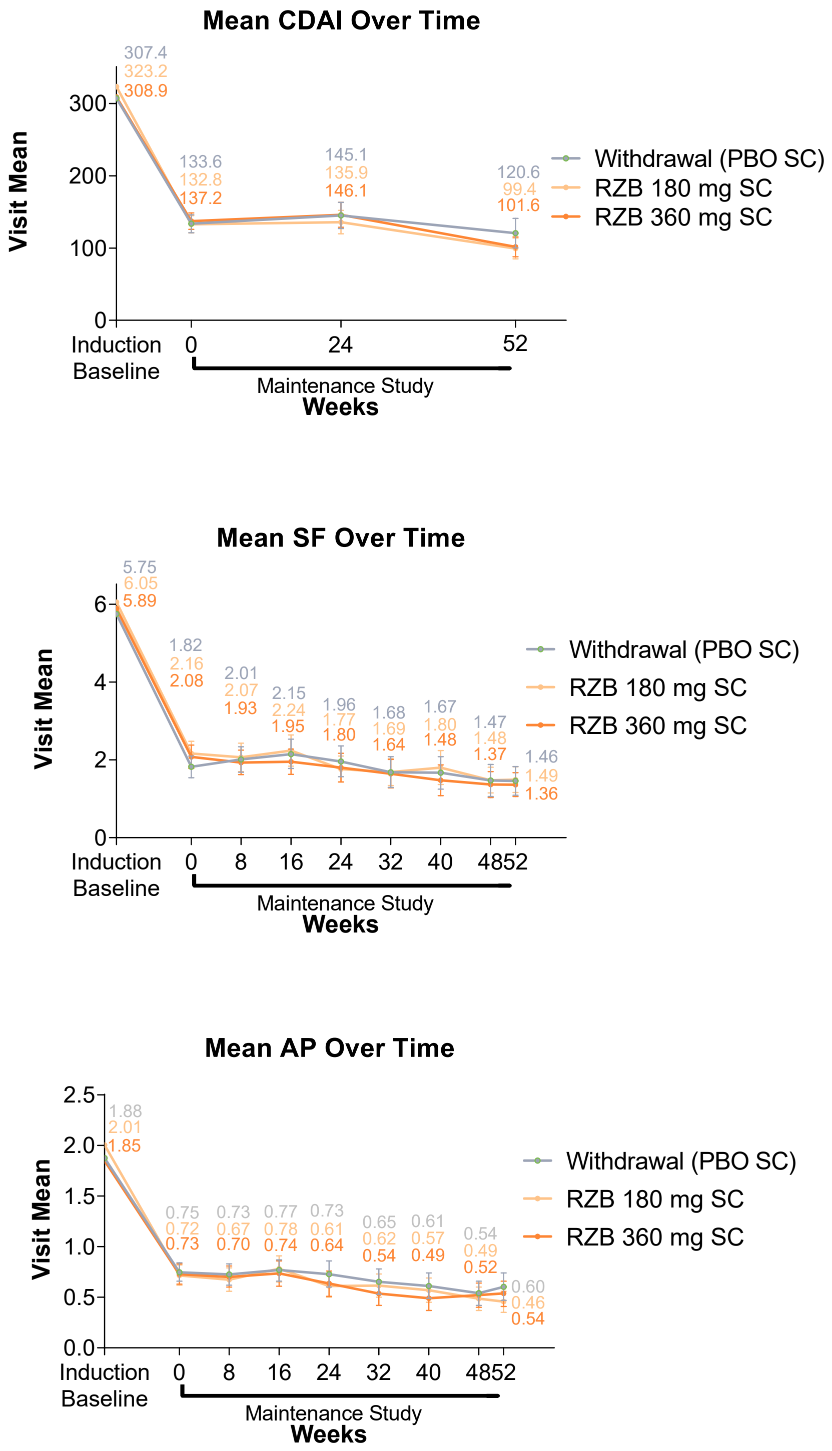

Error bars represent the lower and upper bounds of the 95% confidence interval.
AP, abdominal pain; CDAI, Crohn's Disease Activity Index; PBO, placebo;
RZB, risankizumab, SC, subcutaneous; SF, stool frequency.

Supplementary Figure 6. Clinical remission over time in the maintenance study.

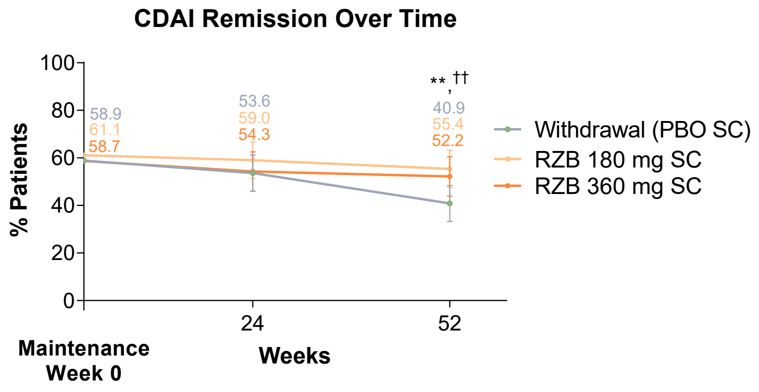

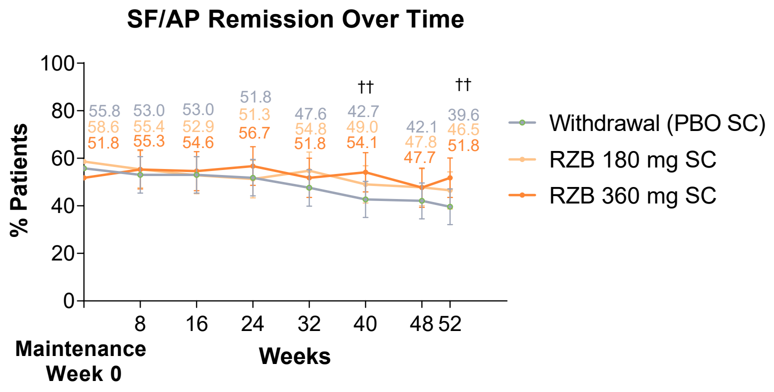

**p ≤0.01 RZB 180 mg SC vs PBO; ††p ≤0.01 RZB 360 mg SC vs PBO. Error bars represent the lower and upper bounds of the 95% confidence interval. AP, abdominal pain; CDAI, Crohn's Disease Activity Index; PBO, placebo; RZB, risankizumab, SC, subcutaneous; SF, stool frequency.
